# 

**Published:** 1907-06

**Authors:** 


					CONTENTS.
Page
Obituary Notice?
Edward Markham Skerrht, M.D. Lond., P.R.C.P.
97
Cases Illustrating the More Unusual Complications of Pneumonia.
By J. Michell Clarke, M.A., M.D. Cantab., F.R.C.P.
io8
The Value of Compression of the Aorta in the Treatment of
Post-Partum Hemorrhage.
By F. Percy Elliott, M.B. Aberd.
Eighty Cases of Lupus Vulgaris.
By W. Kenneth Wills, M.D.Cantab.
127
A Case of Narcolepsy.
By Bertram M. H. Rogers, M.D. Oxon.
144
Tumours and Tubercle in Monkeys.
By W. Roger Williams,
F.R.C.S.
149
Progress of the Medical Sciences :
Medicine.
George Parker, M.D., M.A. Cantab.
152
Surgery.
C. A. Morton
156
Obstetrics.
Walter C. Swayne, M.D. Lond.
162
Reviews of Books
i68
Editorial Notes
177
Notes on Preparations for the Sick
i8o
The Library of the Bristol Medico-Chirurgical Society   182
Meetings of Societies
i?5
Local Medical Notes
i87

				

